# *Klebsiella pneumoniae* and pyogenic liver abscess: Emerging clinical threats, virulence mechanisms and therapeutic strategies (Review)

**DOI:** 10.3892/mmr.2026.13800

**Published:** 2026-01-13

**Authors:** Gulam Mustafa Hasan, Taj Mohammad, Sobia Zaidi, Anas Shamsi, Sukhwinder Singh Sohal, Md. Imtaiyaz Hassan

**Affiliations:** 1 Department of Basic Medical Science, College of Medicine, Prince Sattam Bin Abdulaziz University, Al-Kharj 11942, Saudi Arabia; 2 Centre for Interdisciplinary Research in Basic Sciences, Jamia Millia Islamia, New Delhi 110025, India; 3 Department of Biomedical Sciences, Heritage College of Osteopathic Medicine, Ohio University, Athens, OH 45701, USA; 4 Centre of Medical and Bio-Allied Health Sciences Research, Ajman University, Ajman P.O. Box 346, United Arab Emirates; 5 Department of Laboratory Medicine, School of Health Sciences, College of Health and Medicine, University of Tasmania, Launceston, TAS 7250, Australia

**Keywords:** *Klebsiella pneumoniae*, hypervirulent, pyogenic liver abscess, multidrug resistance, antimicrobial resistance, therapeutic strategies

## Abstract

*Klebsiella pneumoniae* has emerged as a leading cause of pyogenic liver abscess (PLA), driven by hypervirulent and multidrug-resistant (MDR) strains that pose major diagnostic and therapeutic challenges. This organism exhibits extensive capsular diversity (K1-K80), with serotypes K1, K2, K5, K20, K54 and K57 being the most associated with invasive infections and severe clinical outcomes. Increasing convergence between hypervirulence and MDR determinants threatens effective management worldwide. Pharmacological and safety limitations of current antibiotics, including nephrotoxicity of colistin, hepatotoxicity of tigecycline and poor drug penetration into abscess cavities, further complicate treatment and encourage exploration of non-traditional strategies such as anti-virulence or immunomodulatory approaches. Recent advancements in rapid diagnostic tools such as metagenomic sequencing, MALDI-TOF and point-of-care PCR assays offer promising prospects for early detection and antimicrobial optimization. Pharmacokinetic challenges at the abscess site and the emergence of hybrid hvKp-MDR strains emphasize the urgency of precision-guided therapy and robust global surveillance. *K. pneumoniae-*associated PLA thus represents an evolving global health threat and understanding serotype diversity, antibiotic limitations and diagnostic innovations is essential for developing more effective preventive and therapeutic strategies. The present review provides current insights into the epidemiology, pathogenesis and therapeutic challenges of *K. pneumoniae*-associated PLA, while highlighting translational opportunities and research priorities to counter the escalating dual threat of hypervirulence and resistance.

## 1. Introduction

*Klebsiella pneumoniae* is a Gram-negative, encapsulated, facultative anaerobic bacillus that has conventionally been regarded as an opportunistic cause of nosocomial infections, such as pneumonia, urinary tract infections and bacteremia (1). However, the clinical image of this organism has entirely changed over the past 30 years (2). A new clinical syndrome, community-acquired pyogenic liver abscess (PLA), mainly due to hypervirulent *K. pneumoniae* (hvKp), was first noted in Taiwan in the 1980s (3). As a result, hvKp has spread worldwide and is now considered a significant public health threat (4,5). hvKp strains can cause severe, invasive disease in otherwise healthy hosts, unlike classical *K. pneumoniae* (cKp), which is typically isolated from immunocompromised patients in healthcare settings (6). Monomicrobial liver abscess, traditionally associated with capsular serotypes K1 and K2 [such as sequence type (ST) 23 and ST65], and metastatic complications such as endophthalmitis, meningitis, septic pulmonary emboli and necrotizing fasciitis, are clinical hallmarks (7). This shift reflects how a previously opportunistic organism has evolved to cause invasive disease even in healthy hosts (8).

*K. pneumoniae* expresses a wide range of capsular (K) types, currently described as K1 through K80, encoded by distinct cps loci (9). Although >80 K types are recognized, certain serotypes are repeatedly linked to invasive disease. K1 and K2 remain most strongly associated with PLA and metastatic complications; additional serotypes reported from severe or invasive infections include K5, K20, K54 and K57 (10). These serotypes frequently co-associate with key virulence loci such as rmpA/rmpA2 and the aerobactin locus and characteristic sequence types such as ST23 with K1 and ST65 with K2, explaining their propensity for bloodstream invasion and metastatic spread (11). As this issue escalates, the spread of multidrug resistance (MDR) in *K. pneumoniae* continues to intensify, driven by extended-spectrum β-lactamase (ESBL) production and, even more concerning, resistance to carbapenems mediated by enzymes such as *K. pneumoniae* carbapenemase (KPC) (12), New Delhi metallo-β-lactamase (NDM) (13) and OXA-48 carbapenemase (14). Although hvKp strains are generally susceptible to most antibiotics, worrisome cases have been reported of strains carrying both hypervirulence plasmids and MDR determinants (15). The convergence of hypervirulence and antimicrobial resistance represents an emerging global health threat, leading to a critical therapeutic gap with few effective treatment options available (16). PLA due to *K. pneumoniae* differs from other abscesses, such as polymicrobial or enteric abscesses (17).

Historically, the majority of liver abscesses were secondary infections (such as to biliary obstruction or intra-abdominal sepsis) and are typically polymicrobial (18). However, a recent study also highlighted gastrointestinal colonization and translocation as essential portals of entry, even in cryptogenic cases (19). Another example is the pathogenesis of *K. pneumoniae* where a variety of specialized virulence factors cooperate (20). A hypermucoviscous polysaccharide capsule protects bacteria from phagocytosis, iron-uptake siderophore systems sequestering iron from the environmentally restrictive conditions of the mammalian host [such as aerobactin, salmochelin, yersiniabactin (ybt) and enterobactin], colonization factors (such as fimbriae and pili) and lipopolysaccharide (LPS), which enables protection from complement-mediated killing (21). These mechanisms together result in *K. pneumoniae* invasion and traversal across the gut barrier, subsequent colonization of liver parenchyma, evasion of host immunity and dissemination to remote organs (22).

The management of PLA typically involves a combination of antimicrobial therapy and guided percutaneous drainage of the abscess, based on clinical grounds (19). However, the emerging problem of antimicrobial resistance, in conjunction with the invasive capacity of hvKp, challenges treatment success and leads to higher morbidity and mortality (4). In addition, there is currently no licensed vaccine or approved anti-virulence therapy available for treating this pathogen, emphasizing the need for new approaches (23,24). In the present review a comprehensive synthesis of the current knowledge of *K. pneumoniae*-associated PLA is provided. The present review begins with an overview of the epidemiology and clinical importance of these emerging syndromes and summarizes virulence mechanisms and host-pathogen interactions. Then, trends in antimicrobial resistance are discussed, a narrative review of current therapeutic approaches is provided and new concepts such as anti-virulence therapies, immunotherapies and bacteriophage therapy are introduced. Lastly, translational research opportunities and future hurdles related to combating the combined threat of hypervirulence and resistance are highlighted.

## 2. Epidemiology and emerging clinical threats

### Global burden of K. pneumoniae infections

*K. pneumoniae* is one of the most frequently isolated Gram-negative pathogens worldwide and the World Health Organization has designated this bacterial species as a ‘critical priority pathogen’ due to the dual MDR and hypervirulence threat it poses (25). cKp has been a long-standing pathogen of importance in the healthcare setting, having been implicated in ventilator-associated pneumonia, catheter-associated urinary tract infections, intra-abdominal infections and bloodstream infections (26). *K. pneumoniae* has become the focus of numerous challenging nosocomial epidemics as it possesses the unique ability to rapidly capture resistance determinants, ESBLs and carbapenemases (14). At the same time, hvKp is a relatively recently recognized pathotype and its emergence has significantly broadened the clinical spectrum of *K. pneumoniae* infections (6).

hvKp was first identified in the 1980s in Taiwan and it has become an international concern as a source of both community-acquired and virulent invasive syndromes in both immunocompromised and healthy hosts (4). Of these, PLA is the most typical and clinically meaningful manifestation. Recent surveillance data derived from multinational hospital-based surveillance networks and national reporting systems from routine culture and genomic analyses collected between 2020 and 2024 documented sporadic autochthonous hvKp-PLA cases outside Asia, including South America and Africa, suggesting that the global spread may be underestimated due to underdiagnosis (4).

#### PLA as a distinct clinical entity

hvKp-associated PLA differs significantly from classical polymicrobial liver abscesses (27). Traditionally, the majority of liver abscesses develop as polymicrobial infections secondary to biliary tract disease, appendicitis or intra-abdominal sepsis (18). By contrast, hvKp-PLA is generally monomicrobial and more often cryptogenic, without an evident underlying hepatobiliary abnormality or secondary cause (3). Capsular serotypes K1 and K2 account for the majority of cases and sequence types ST23, ST65 and related clones are predominant (9). Patients classically present with acute fever, localized epigastric or right-upper-quadrant abdominal pain, along with evidence of systemic inflammation (28). A hallmark of hvKp is its propensity for bacteremia with metastatic spread, most commonly presenting as endogenous endophthalmitis, central nervous system infections such as meningitis or brain abscess, or pulmonary complications such as septic emboli or pneumonia (29). Overall, hvKp-PLA has lower mortality rates than polymicrobial abscesses if detected and treated early; however, complications, including severe morbidity, are common when either diagnosis or drainage is delayed (19).

### Risk factors and host predispositions

Although hvKp can cause disease in healthy hosts, several risk factors significantly predispose to disease (4). The most common and well-recognized predisposing factor is diabetes mellitus; nearly all cohorts report greater than half of PLA patients with underlying diabetes (30). Experimental data indicate that hyperglycemia impairs neutrophil chemotaxis and phagocytic function, leading to impaired host defence (31). Chronic liver disease, cirrhosis, malignancy and other immunosuppressive therapies are some other factors that can contribute to vulnerability (32). However, hvKp is characterized by its capacity to cause severe disease in hosts without obvious comorbidities, contrasting with cKp infections, and challenging long-held assumptions about host susceptibility (6). While hvKp can produce invasive disease in healthy individuals, a number of comorbidities confer a marked increase in risk, including diabetes mellitus (Table I).

Although hvKp can infect otherwise healthy individuals, epidemiological studies consistently identify certain patient populations as being at higher risk of developing PLA and other severe complications (4). These include individuals with diabetes mellitus, both type 1 and type 2, where impaired neutrophil chemotaxis and phagocytic function contribute to increased susceptibility; patients with chronic liver disease or cirrhosis, in whom hepatic immune dysfunction and portal hypertension facilitate bacterial dissemination; and immunocompromised individuals receiving systemic corticosteroids, cytotoxic chemotherapy or biological immune modulators (31). Increased risk is also observed among patients with malignancies, those with indwelling devices or a history of abdominal instrumentation that disrupts mucosal barriers, as well as in the elderly and neonates, where host defence mechanisms are often diminished (32). Notably, hvKp can occasionally cause severe infections even in young, previously healthy individuals, emphasizing that the absence of comorbidities does not preclude the risk of invasive disease.

### Geographical trends and strain distribution

Epidemiology reveals a precise geographical distribution associated with hvKp PLA (4). In East Asia, especially Taiwan, China, South Korea and Singapore, hvKp has become the predominant agent of community-acquired liver abscess (33). Initial cases described outside Asia were predominantly associated with immigrants or travellers (34). However, more recently, autochthonous infections have been documented in Europe and North America, indicating that hvKp is now establishing endemic transmission in some non-Asian regions (35). As for South Asia, the Middle East and Africa, fewer reports from these regions exist; however, this is most likely due to a lack of recognition rather than an absence (36). Surveillance from developing nations, notably parts of South Asia, the Middle East and selected African countries, indicates a complex image: A number of reports show both classical MDR *K. pneumoniae* and hvKp lineages, sometimes in mixed circulation (35). In South Asia, for example, hvKp lineages (such as ST23) have been reported alongside ST11/ST15 clones carrying NDM or other carbapenemases, creating regional hotspots for convergence events (36). Resource and capacity limitations for routine molecular surveillance likely understate true incidence in many low- and middle-income countries (LMICs).

Accordingly, terms such as ‘global trends’ should be interpreted as geographically heterogeneous, with high burdens of hvKp-PLA reported in East Asia, rising autochthonous cases in high-income regions, and concerning reports of resistance-associated hvKp from LMICs where surveillance is improving (37). The lineage types involved in PLA are also well-characterized using molecular epidemiology, with ST23 (typically K1), ST65, ST86 and ST375 being the most prominent lineages associated with PLA (10). The emergence of hvKp with its virulence plasmids harbouring the rmpA gene and siderophore biosynthesis clusters on different genetic backgrounds indicates the genomic flexibility of hvKp for horizontal gene transfer and genetic adaptation (11). Recent genomic epidemiology studies also highlight convergence events involving ST11 and ST15 lineages, which carry both carbapenem resistance and virulence plasmids (36,37). Notably, different geographical patterns have been documented, with hvKp first described in East Asia but now found worldwide (Table II).

### Convergence of hypervirulence and antimicrobial resistance

Between the two critical epidemiological developments, the global rise of hypervirulent hvKp strains and the rapid spread of MDR among classical *K. pneumoniae*, the most worrying is the convergence of hypervirulence and MDR. Despite their invasive behaviour, hvKp strains were susceptible to most antibiotics for a number of years, which allowed for high treatment effectiveness (4). In comparison, classical strains rapidly acquired resistance genes, such as ESBLs and carbapenemases, including KPC, NDM and OXA-48 (38). However, researchers recently reported hvKp isolates with those resistance genes in China, India, Europe and North America (4,39). These strains represent a worst-case scenario, as they combine extensive drug resistance with high metastatic potential (40). Molecular studies have shown that homologous mobile plasmids constitute a significant part of this phenomenon, transferring resistance genes to hvKp lineages or virulence genes to MDR classical strains (41,42). However, the emergence of carbapenem-resistant hypervirulent *K. pneumoniae* (CR-hvKp) has raised an urgent need for surveillance and containment (42).

### Clinical and public health implications

The changing epidemiology of hvKp-associated PLA has important implications for clinicians and public health. The heterogeneous virulence of hvKp complicates diagnosis for clinicians, as distinguishing hvKp from cKp is not straightforward in routine clinical settings. While differential testing by the hypermucoviscosity ‘string test’ is widely used, it is not sensitive or specific and advanced molecular assays are still limited in clinical routine (11). Rapid molecular assays targeting aerobactin (iuc locus) or rmpA genes have shown higher accuracy but remain largely confined to research settings (43). The emergence of hvKp with reduced susceptibility to the standard antibiotic regimen (including image-guided drainage) is progressively leading to therapeutic dilemmas (44). However, outside East Asia, surveillance is often opportunistic, and the actual global burden is likely underestimated due to underdiagnosis and limited surveillance infrastructure outside Asia. Given its ability to produce life-threatening, invasive disease in patients who were previously healthy, coupled with its rapid acquisition of antimicrobial resistance, hvKp has the potential to become a pandemic organism. The global emergence of hvKp exemplifies how a classical opportunistic pathogen has evolved from being community and healthcare-associated to becoming a significant threat with consequences far beyond liver abscess.

## 3. Pathogenesis and virulence mechanisms

The liver abscesses caused by hvKp result from multiple virulence mechanisms (33). These virulence determinants, which include polysaccharide capsule, siderophore systems, adhesins, LPS modifications and plasmid-encoded factors, exhibit 2- to 1,000-fold upregulated expression *in vivo* (45). This section will evaluate these mechanisms in-depth.

### Capsule and hypermucoviscosity phenotype

The polysaccharide capsule may be the most unique virulence factor of *K. pneumoniae* (46). Hypermucoviscous capsule production (commonly evaluated with the ‘string test’) is one of the markers that is highly associated with hvKp (47). Capsule regulators rmpA and rmpA2, encoded on large virulence plasmids, primarily control this phenotype (48). K1 and K2 serotypes typically form very mucoid capsules that are resistant to phagocytosis and the complement system. The hypermucoviscous phenotype is lost in rmpA/rmpA2 mutants and the virulence of these mutants is impaired in animal models (49). Additionally, the capsule prevents neutrophil phagocytosis and protects the organism from recognition by antibodies (50). A recent narrative review showed that sequencing studies have identified structural differences in capsular operons between hvKp and cKp, which may contribute to the enhanced virulence of hvKp (11).

### Siderophore-mediated iron acquisition

Although iron is a mineral that bacteria need to grow, it is locked away by host proteins, including transferrin and lactoferrin (51). The high affinity siderophores produced by *K. pneumoniae* enable it to bypass this nutritional immunity. Hypervirulent strains encode multiple iron acquisition systems (such as enterobactin, ybt, salmochelin and aerobactin), with aerobactin being nearly universal and the most critical for hvKp virulence in PLA (52). Siderophores mediate bacterial expansion in liver parenchyma and blood by rapidly scavenging iron from the host environment (53). Besides iron acquisition, certain siderophores serve as immunomodulators, leading to oxidative stress and a diminished responses from neutrophils (54). The redundancy affords hvKp with a significant evolutionary advantage in a host environment where nutrients are often limited. Recent studies also suggest that aerobactin expression correlates with increased metastatic potential and poorer clinical outcomes (33,55).

### Fimbrial adhesins and colonization

Fimbriae play a crucial role in the initial attachment to host tissues (22). At present, type 1 and type 3 fimbriae in *K. pneumoniae* have been best characterized. Type 1 fimbriae allow binding to mannose-containing receptors on epithelial cells, whereas type 3 fimbriae promote biofilm formation individually or on biotic and abiotic surfaces, including medical devices. Fimbrial adhesins have been shown to promote gastrointestinal colonization of bacteria and increase the likelihood of translocating microbes through the intestinal mucosa into the portal circulation, potentially facilitating the formation of liver abscesses (22). Once in the liver, fimbriae facilitate adhesion to hepatic tissue, allowing for persistence and the formation of abscesses. Together with capsule and siderophores, these adhesive structures enable hvKp to establish a robust infection niche. *K. pneumoniae* employs a wide array of virulence factors, from capsule, siderophores, fimbrial adhesins and LPSs (Table III).

### LPS and immune evasion

The LPS of *K. pneumoniae* also protects bacteria from complement-mediated lysis, thereby contributing to its pathogenesis (56). Various O-antigen structures of LPS directly impact serum resistance and binding by host pattern recognition receptors (PRRs) (57). HvKp strains are proposed to have adapted LPS that confer serum resistance, with the capsule playing a protective role simultaneously. Beyond structural defence, LPS is a potent inducer of inflammation, driving hepatocellular injury and systemic inflammatory responses that exacerbate PLA severity (58).

### Virulence plasmids and mobile genetic elements

Most hvKp virulence factors are encoded on large virulence plasmids (200–220 kb) (59). Such plasmids encode genes for rmpA/rmpA2 and the aerobactin (iuc locus), salmochelin (iro locus) and other accessory factors (60). This is especially concerning since their horizontal mobility enables transfer of hypervirulence traits to classical MDR strains, producing the so-called convergence phenotype. In addition, hvKp virulence is further expanded by chromosomal pathogenicity islands, including those coding for ybt and colibactin (clb), which provide unique mechanisms for iron acquisition and genotoxicity, respectively (6). A study has linked clb production to colorectal carcinogenesis, highlighting potential long-term sequelae of hvKp colonization (61). The adaptability and clinical success of hvKp stems from the complex interplay among plasmids, pathogenicity islands and host genomic backgrounds.

### Mechanistic basis of liver abscess formation

**Figure 1.**
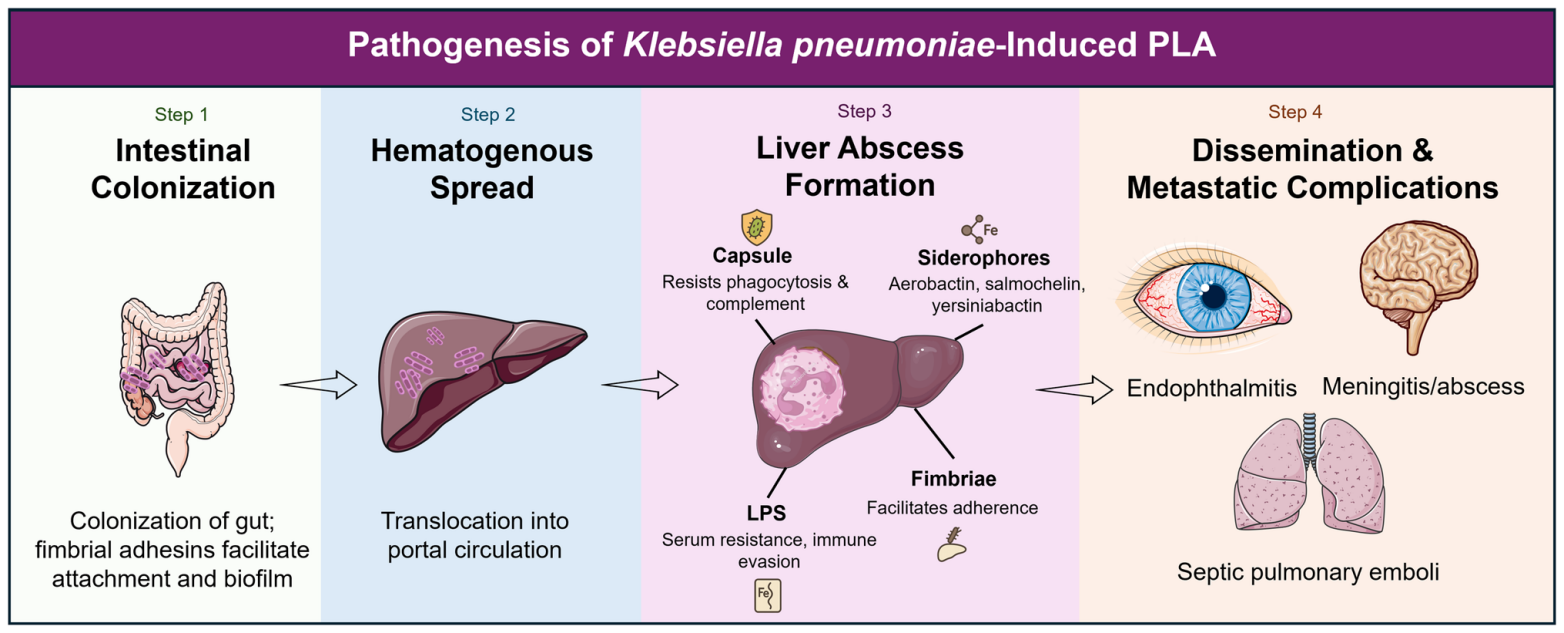
Mechanistic overview of *Klebsiella pneumoniae* pathogenesis leading to pyogenic liver abscess (PLA). The schematic shows gut colonization and translocation, key virulence factors (capsule, siderophores, fimbriae and LPS), bloodstream invasion, hepatic colonization, abscess formation and metastatic dissemination. PLA, pyogenic liver abscess; LPS, lipopolysaccharide.

PLA is the result of a series of microbial-host interactions that lead to disease pathogenesis (17). The initial reservoir is intestinal colonization, followed by bacterial translocation into the portal venous circulation (3). The hvKp capsule and LPS facilitate the avoidance of phagocytic clearance once the bacterium reaches the liver (50). The production of siderophores ensures survival in the iron-limited hepatic microenvironment and fimbrial adhesins facilitate tissue adherence (51). Neutrophil and macrophage recruitment lead to regional inflammation, necrosis and the formation of an abscess cavity (31). By contrast, hvKp liver abscesses are almost always monomicrobial rather than polymicrobial, as is the case for most abscesses caused by anaerobes and enteric flora, emphasizing the intrinsic capacity of the pathogen to establish infection independently (33). The rapidity of metastasis in these patients is therefore closely related to the ability of hvKp to penetrate the bloodstream and disseminate systemically via its capsule and plasmid-borne virulence arsenal. Virulence factors, including capsule, siderophores, fimbrial adhesins and LPS, work together to facilitate colonization of the liver, evade the immune response and promote systemic spread (Fig. 1).

## 4. Host-pathogen interactions and disease manifestation

Colonization of the host by *K. pneumoniae* and subsequent progression to PLA are multifaceted processes involving the interplay of virulence factors and immune responses of the host (22). Such hypervirulent strains appear to have developed mechanisms that enable them to both evade immune clearance and exploit host physiological weaknesses, thereby facilitating invasive disease with metastatic sequelae. This section elaborates on the mechanisms of maintaining virulence while evading both innate and adaptive immunity, ultimately culminating in hepatic invasion, abscess formation and systemic complications.

### Hepatic invasion and abscess formation

*K. pneumoniae* translocates from the gastrointestinal tract and reaches the liver via portal circulation (62). The gastrointestinal mucosa, especially during dysbiosis or increased permeability, serves as a significant reservoir. After entering the portal venous system, hypervirulent strains are not phagocytosed or killed by complement as they possess a thick polysaccharide capsule and a hypermucoid phenotype (63). This allows the bacterium to survive in the bloodstream and seed hepatic tissues. In the liver parenchyma, bacteria attach to hepatocytes and endothelial cells through fimbrial adhesins, and iron acquisition through siderophores promotes bacterial replication in the iron-limited hepatic microenvironment (64). With advancing infection, this leads to local necrosis and tissue liquefaction, promoted by bacterial toxins and host mediators of inflammation. The result is the development of defined abscess cavities filled with pus material, often multiloculated, which may increase in size or number.

### Innate immune responses

Innate immune cells are abundant in the liver, including Kupffer cells, neutrophils and natural killer cells, which form the first line of defence against invading pathogens (65). PRRs recognize bacterial components, such as LPS and capsular polysaccharides, initiating an inflammatory cascade that is associated with cytokine release and the recruitment of neutrophils (66). Nevertheless, hypervirulent *K. pneumoniae* strains evade neutrophil extracellular traps, inhibit complement activation and resist phagocytosis, thereby allowing them to persist in hepatic tissue. Neutrophils are a hallmark of PLA pathophysiology; however, excessive neutrophil accumulation contributes to collateral tissue injury, hepatocyte necrosis and the progression of abscesses (67). Similarly, with the activation of Kupffer cells, pro-inflammatory cytokines are produced, which enhance local inflammation but typically do not clear the pathogen from the liver (68). This inappropriate immune reaction leads to an environment that favours bacterial persistence and abscess maturation.

### Adaptive immune responses

The adaptive immune system plays a crucial role in containing and clearing liver abscesses (33). Antibody-mediated responses against capsular polysaccharides and siderophores enhance opsonophagocytosis, while T cell-mediated immunity contributes to bacterial clearance. However, in individuals with diabetes, chronic liver disease or immunosuppression, adaptive immune responses are impaired, resulting in dysregulated bacterial growth and severe disease (66). The diversity of capsule and antigenic variability makes hypervirulent strains unusually adept at evading antibody-mediated recognition. Additionally, bacterial metabolites and endotoxins may suppress adaptive immunity, thereby prolonging the infection and facilitating its dissemination (66).

### Metastatic infections and extrahepatic complications

A hallmark of hvKp-mediated PLA is its ability to induce metastatic infections at non-contiguous sites (66). Through bacteremia, it can spread to the eyes, central nervous system, lungs and other organs. Endophthalmitis (often leading to irreversible vision loss), meningitis and brain abscesses are among the most devastating complications, associated with high morbidity and mortality (69). These processes are critical mechanisms driving metastatic spread, including complement resistance, neutrophil resistance and vascular invasion. The metabolic flexibility enabled by siderophore systems supports survival in these nutrient-limited niches, such as the vitreous humour and cerebrospinal fluid. The ability to disseminate despite host immune defences underscores the enhanced virulence potential of hvKp compared with cKp.

### Clinical outcomes and prognosis

*K. pneumoniae* PLA can present from simple localized hepatic abscesses with fever and abdominal pain to fulminant sepsis with multi-organ involvement (18). Therapy and prognosis are influenced by three key factors: The immune status of the host, bacterial virulence and the timing of intervention. Older patients, patients with diabetes and patients with underlying liver disease are predisposed towards severe disease and poor outcomes. Patients with resistant infections, metastatic complications or delayed diagnosis experience substantially higher mortality (70). The host-pathogen interactions underlying hvKp-mediated PLA are ultimately defined by a combination of the evasion of the pathogen from the innate and adaptive immunity, the immunological background of the host and the fine line between protective inflammation and harmful immune pathologies (29). Understanding these interactions is essential for designing targeted therapies that bolster host defences while neutralizing bacterial virulence.

## 5. Antimicrobial resistance in K. pneumoniae PLA

*K. pneumoniae* PLA is becoming increasingly challenging to treat due to the widespread emergence of antimicrobial resistance (19). The division between hypervirulent strains, which were initially mostly susceptible to most antibiotics, and classical strains, which were primarily associated with MDR in a nosocomial background, is also no longer absolute. Hypervirulent and resistant strains are increasingly appearing, posing a significant therapeutic challenge (4). This section summarizes current resistance patterns, mechanisms and clinical consequences specific to PLA.

### Global resistance trends

Antimicrobial resistance in *K. pneumoniae* has been reported worldwide, with the highest prevalence observed in regions where PLA is endemic (71). The increase in ESBL production of *K. pneumoniae* is particularly high in East and Southeast Asia, where multidrug class co-resistance is common (72). Carbapenem-resistant *K. pneumoniae* (CRKP) has also emerged globally, driven by carbapenemases such as KPC (prevalent in the Americas and Europe), NDM (dominant in the Indian subcontinent) and OXA-48-like enzymes (common in the Middle East and Europe) (73,74). Resistance genes have begun to emerge in a significant proportion of hvKp isolates associated with liver abscesses (27). The rate surpassed 20% in the networks for ESBL-hvKp-PLA strains in both China and Taiwan between 2020 and 2024 (75). Although fundamentally uncommon, carbapenem resistance is increasingly reported in hvKp-PLA, particularly among healthcare-associated cases or in endemic countries with CRKP (76). A surveillance study has indicated that up to 10–15% of hvKp isolates in China and India now harbour carbapenemase genes, reflecting a rising convergence of resistance and hypervirulence (73).

### Mechanisms of resistance

Resistant mechanisms in *K. pneumoniae* are heterogeneous and often act cooperatively to produce strong MDR profiles (77). The most common mechanism is the production of β-lactamases, including ESBLs and carbapenemases, which inactivate extended-spectrum cephalosporins and carbapenems (78). Resistance to β-lactam class antibiotics typically develops from ESBLs, such as Cefotaximase-Munich (CTX-M) and sulfhydryl variable, and carbapenemases, such as KPC, NDM, OXA-48 and Verona integron-encoded metallo-β-lactamase (79). These enzymes are often accompanied by porin loss (OmpK35/36) and upregulation of efflux pumps, thereby enhancing resistance. Plasmids containing *bla*CTX-M and *bla*KPC are frequently associated with fluoroquinolone and aminoglycoside resistance genes (80). A broader concern regarding salvage therapy is the development of colistin and tigecycline resistance, which can occur via mcr genes or chromosomal alterations in the case of colistin, and through ramR mutations or efflux pumps in the case of tigecycline, in *K. pneumoniae* in general (81). The development of mutations or loss of outer membrane porins, particularly OmpK35 and OmpK36, results in a decrease in drug permeability, thereby increasing the potency of β-lactamase activity (82). Efflux pumps, such as the AcrAB-TolC system, when upregulated, render lower intracellular concentrations of numerous antibiotic classes, which also include fluoroquinolones and tigecycline (83). Aminoglycoside resistance typically arises from enzymatic modification or ribosomal methylation. By contrast, colistin resistance develops through plasmid-borne mcr genes or chromosomal regulatory mutations resulting from chromosomal alterations in the regulatory pathways or the acquisition of plasmid-borne mcr genes (84). This can occur in a single isolate, resulting in organisms that are resistant to nearly all clinically available antibiotics. Resistance mechanisms, including β-lactamase production, porin loss and efflux pumps, act synergistically (Table IV).

### Convergence of hypervirulence and MDR

**Figure 2.**
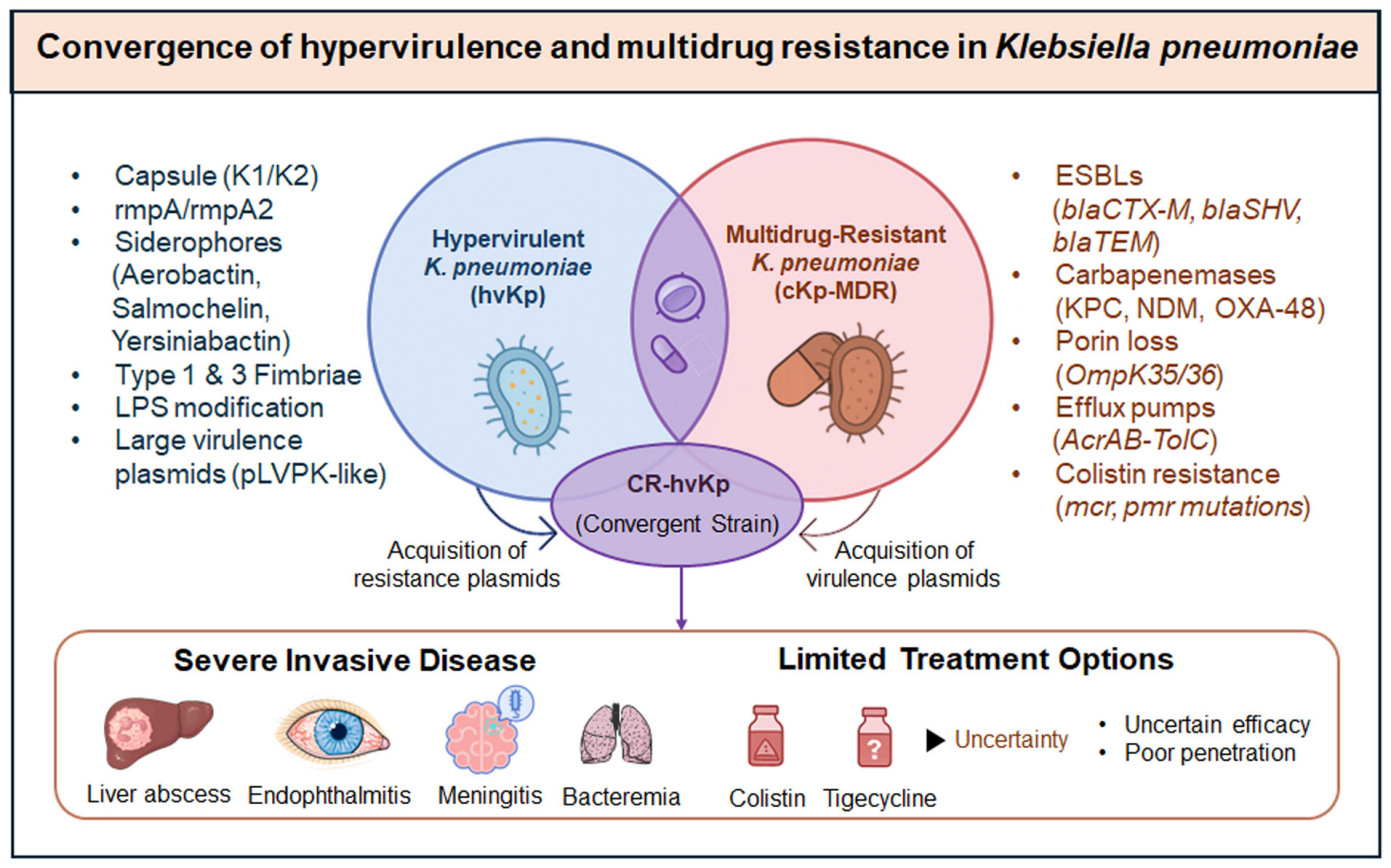
Convergence of hypervirulence and multidrug resistance in *Klebsiella pneumoniae*: an evolving global threat. The schematic contrasts the historical separation of classical (cKp) and hypervirulent (hvKp) lineages with recent plasmid-mediated convergence events that produce carbapenem-resistant hypervirulent strains. These hybrid strains represent a critical global health threat due to invasive disease and limited therapeutic options. LPS, lipopolysaccharide; pLVPK, large virulence plasmid of *Klebsiella pneumoniae*; MDR, multidrug resistance; ESBLs, extended-spectrum β-lactamases; KPC, Klebsiella pneumon*iae* carbapenemase; NDM, New Delhi metallo-β-lactamase; OXA-48, oxacillinase-48.

A growing and serious threat is the convergence of hypervirulence with MDR (6,25). Hypervirulent strains, known for causing severe disease with metastatic complications, were long considered manageable due to their susceptibility to antibiotics, providing clinicians with an essential therapeutic window. However, this advantage is rapidly diminishing as hypervirulent strains acquire plasmids carrying ESBLs and carbapenemases (35). This convergence is not an isolated event, but a global problem, as evidenced by reports of outbreaks caused by carbapenem-resistant hypervirulent strains. hvKp and MDR-cKp lineages have historically circulated separately, but recent plasmid exchanges are blurring this distinction (85). Nonetheless, recent surveillance data have described hvKp strains, often ST23 or ST11 clones, that have acquired ESBL or carbapenemase genes on plasmids (86,87). The transfer of plasmids and the integration of resistance loci into the chromosomal genome drive this convergence. Some examples include hvKp isolates associated with *bla*NDM or *bla*KPC, which have been reported from China and India, with some found in the absence of PLA in Europe. The recent rise of strains with simultaneous hypervirulent (K1/K2 capsule, aerobactin) and carbapenem-resistant traits has been coined as ‘hypervirulent carbapenem-resistant *K. pneumoniae* (hv-CRKP)’ with global alerts issued (10). Such convergence is illustrated in Fig. 2, which contrasts the historical separation of cKp and hvKp, as well as the recent emergence of CR-hvKp due to the plasmid-mediated exchange of resistance or virulence determinants (88). These hybrid strains exhibit the worst aspects of both phenotypes and pose a significant clinical and public health challenge.

### Clinical implications of resistance in liver abscess

Antimicrobial resistance notably affects the clinical presentation and treatment of liver abscesses (33). Empirical therapy with cephalosporins often fails in areas with high ESBL prevalence, resulting in delayed bacterial clearance and adverse outcomes (33). This leaves few options, especially with carbapenem resistance, frequently restricting clinicians to last-line drugs such as colistin and tigecycline. However, both agents have limitations, such as poor penetration into abscess spaces, toxicity and variable efficacy (33). Consequently, patients with resistant infections may require more extended hospital stays, combination regimens with complex and challenging combinations and invasive procedures. Outcomes are particularly poor in hvKp strains that are also multidrug resistant, as they are strongly associated with bacteremia, septic shock, metastatic complications and high mortality.

### Limitations of current antibiotic therapies

The treatment of *K. pneumoniae* PLA with conventional antibiotic therapy has become less reliable in recent years (33). The widespread resistance against once highly effective third-generation cephalosporins and carbapenems is undermining this progress (89). Target-site mutations and efflux contribute to fluoroquinolone resistance, and aminoglycosides are destroyed by enzymatic modification. Colistin, otherwise a last-line antibiotic, faces increasing resistance, as well as nephrotoxicity and uncertain effects in liver abscesses (45). In addition to emerging resistance, several commonly used agents have clinically important adverse effects and pharmacological limitations that influence therapeutic decision-making in PLA. Colistin is nephrotoxic and its pharmacodynamics in poorly vascularized abscess cavities are uncertain; renal adverse effects often limit dose escalation (90). Tigecycline has poor serum concentrations, can cause significant gastrointestinal side effects and has been associated with increased mortality signals in a meta-analysis; its penetration into abscess cavities is variable and often suboptimal (91).

Aminoglycosides have nephro- and ototoxicity risks that restrict prolonged use, and β-lactams, including many third-generation cephalosporins and even carbapenems, may inadequately penetrate the necrotic centre of large, poorly vascularized abscesses (92). These adverse events and pharmacokinetic limitations, such as reduced vascular delivery, high protein binding and sequestration in necrotic material, help explain treatment failures and motivate alternative local or adjunctive delivery strategies, such as intra-cavitary instillation and drug-eluting devices (93). A number of antibiotics have pharmacokinetics that limit their penetration into abscess cavities, thereby complicating complete eradication (93). This bottleneck highlights the need for alternative strategies that target both antimicrobial resistance mechanisms and the pathogenic potential of hypervirulent strains, beyond classical antibiotics.

## 6. Current therapeutic approaches

Management of *K. pneumoniae* PLA involves a combination of empirical antimicrobial therapy and interventional procedures that target abscess drainage. Although the early initiation of antibiotics is crucial, the rising prevalence of antimicrobial resistance complicates the choice of empirical regimens. Source control, which is often required for definitive cure, is also obtained with the aid of interventional radiology and surgical procedures.

### Antimicrobial therapy

Empirical antibiotic therapy for PLA is initiated as soon as the diagnosis is confirmed, with adjustments made based on culture and susceptibility results (94). Third-generation cephalosporins, such as ceftriaxone or ceftazidime, have historically been the preferred choice due to their effectiveness against susceptible strains and adequate biliary penetration (95). However, in areas of high ESBL prevalence, carbapenems have frequently been used as first-line therapy. Carbapenem resistance has further complicated management, leaving tigecycline, colistin and, in some instances, aminoglycosides as the only alternative agents (73). Combination therapy is often employed in severe or resistant infections, although supporting evidence remains limited. Carbapenems may also be used in combination with aminoglycosides or fluoroquinolones to facilitate bacterial clearance, although the evidence for these combination regimens is mixed.

For MDR or extensively drug-resistant strains, salvage therapies such as colistin-tigecycline combinations are occasionally used, although concerns are present regarding efficacy and toxicity (95). The length of antimicrobial treatment ranges from weeks to months, depending upon clinical condition, radiological resolution of the abscess and clearance of microbiological measures. The pharmacokinetics of antimicrobials represent a crucial aspect of managing PLA (96). Therapeutic concentrations of antibiotics are not only in the blood but also in hepatic tissue and the abscess cavity. The encapsulated nature of liver abscesses and necrotic material can limit the penetration of drugs requiring long-course treatment, and in most cases, also drainage procedures (97).

The efficacy of antibiotic therapy in PLA depends not only on systemic susceptibility profiles but also on achieving effective drug concentrations within the abscess cavity (19). Several pathophysiological factors can markedly reduce intra-cavitary antibiotic exposure, including poor vascularity and the presence of necrotic debris that impede drug delivery, the high protein and lipid content of abscess material can bind and inactivate antimicrobials and acidic or hypoxic microenvironments that alter drug activity (96). Although β-lactams generally attain favourable biliary and hepatic tissue concentrations, their penetration into poorly perfused abscess cores may remain suboptimal (97). Lipophilic agents such as, tigecycline may distribute to hepatic tissue but often display low serum concentrations; conversely, aminoglycosides have limited penetration into abscess fluid (91,92). Local pharmacokinetic studies are limited, but measured abscess/plasma ratios have shown substantial variability across agents and individual patients (92). These limitations provide a rationale for local delivery approaches such as intra-cavitary instillation, catheter-directed infusion or drug-eluting beads and for tailoring systemic therapy guided by pharmacodynamic/pharmacokinetic principles when possible.

### Interventional management

Besides antibiotics, drainage of the abscess is an essential form of source control (98). Ultrasonography or computed tomography-guided percutaneous catheter drainage is the standard of care for the majority of cases and has replaced open surgical approaches in most cases (99). Drainage reduces bacterial load and relieves pressure from expanding cavities, thereby enhancing antibiotic penetration and alleviating mass effect. The drainage will be based on the size/number and location as well as the clinical condition of the patient. However, small abscesses of <3 cm may be managed with antibiotics alone and close follow-up (19). Percutaneous drainage is typically needed for larger abscesses, multiloculated cavities or abscesses that show a poor response to medical therapy. Surgical drainage is reserved for complicated cases, including multiloculated abscesses, failed percutaneous drainage, rupture or perforation with peritonitis (100). Both diagnosis and management are imaging centred. Ultrasonography is frequently used for initial diagnosis and to guide drainage procedures, while computed tomography has higher sensitivity for diagnosing abscess formation, especially in cases of multiple or small lesions. Imaging follow-up is also necessary to assess both response to treatment and to identify recurrence.

### Integrated management considerations

Prompt intervention combined with appropriate antibiotics is the optimal treatment for *K. pneumoniae* PLA, which requires a multidisciplinary approach (101). Glycaemic control and supportive care are crucial for managing diabetes and other comorbidities, as they significantly impact outcomes in such scenarios (102). Management also focuses on preventing complications such as sepsis, metastatic spread and organ failure. The identification of resistant organisms and the prompt modification of antimicrobial therapy play crucial roles, with delays in effective treatment being closely linked to patient outcomes. Despite advances in diagnostic imaging and interventional radiology, morbidity and mortality remain high, especially in MDR infections and hvKp strains with metastatic spread. These limitations underscore the critical need for innovative treatment modalities that move beyond standard antibiotics and drainage steps, setting the stage for the forthcoming innovative strategies addressed in the next section.

## 7. Emerging and experimental therapeutic strategies

The limitations of traditional antibiotic treatments, combined with the notable increase in the incidence of MDR strains of *K. pneumoniae*, have sparked renewed interest in novel and complementary therapeutic approaches (101). These emerging approaches aim not only to circumvent resistance but also to neutralize virulence and strengthen host defences (103). Other antimicrobial approaches, such as exploring new antimicrobials and anti-virulence agents, immunotherapies and biological modalities, including bacteriophage and microbiome interventions, remain under consideration for the treatment of PLA due to *K. pneumoniae* (14).

### Novel antibiotics and combination therapies

Novel β-lactam/β-lactamase inhibitor combinations and non-β-lactam antibiotics have been developed in response to the global spread of CRKP (104). For KPC-producing strains, ceftazidime-avibactam and meropenem-vaborbactam target KPC, and imipenem-relebactam extends the coverage against some OXA-48 producers (38). Cefiderocol, a siderophore-cephalosporin, demonstrates potent activity against carbapenemase-producing bacteria via iron uptake pathways (103). Nonetheless, resistance is reported to emerge to ceftazidime-avibactam in KPC-CRKP, warranting stewardship. Other options include newer tetracycline derivatives (such as eravacycline) and aminoglycoside formulations (such as plazomicin), for which limited experience is available in PLA.

### Anti-virulence approaches

Considering that hypervirulent strains do not merely cause a more severe disease by being more resistant, but have evolved a different set of pathogenic weapons, developing therapies to neutralize these virulence factors provides an appealing approach. Inhibition of capsule biosynthesis has been explored to destabilize the protective layer that protects bacteria from the host immune system (66). Agents targeting siderophore systems prevent bacterial access to iron, a key nutrient for survival and dissemination. Additionally, the disruption of biofilm formation and quorum sensing, which promote persistence and help avoid host responses, are also promising targets. Although they may not eradicate bacteria directly, anti-virulence therapies can render pathogens more susceptible to immune clearance, thereby enhancing the efficacy of companion antibiotics.

### Immunotherapy and vaccine development

Another promising direction involves immunological strategies (50). An animal study has demonstrated that monoclonal antibodies against capsular polysaccharides, LPSs or siderophores enhance opsonophagocytosis and provide passive protection (105). K1 and K2 serotypes, which are most often implicated in hypervirulent liver abscess strains, are primary targets of whole-cell and subunit vaccines being developed as part of vaccination efforts (106). Preclinical and early clinical studies suggest that immunization may provide long-lasting protection in at-risk populations, such as individuals with diabetes or chronic liver disease; however, no vaccine has yet been licensed (23,106). Phase I/II trials of K1/K2 capsule-based vaccines and siderophore-conjugate vaccines are ongoing, but clinical translation remains pending (107). Host-directed immunotherapies target not only the pathogen but also aim to augment the innate immune responses of the host and correct the disturbed immunity, thereby tipping the host-pathogen balance in favour of bacterial clearance.

### Bacteriophage therapy and phage-derived enzymes

Bacteriophage therapy has resurfaced as a potential treatment for MDR *K. pneumoniae* infections, similar to the scope of PLA (108). Natural phages against hypervirulent strains have been effective in preclinical studies, rapidly lysing bacteria resistant to antibiotics (109,110). Phage cocktails are being explored to mitigate resistance development, with early clinical trials now underway (110). Moreover, phage-derived lysins, enzymes that degrade bacterial cell walls, also represent a promising strategy with broad lytic activity and the ability to work synergistically with antibiotics. Although phage-host specificity, regulatory considerations and *in vivo* efficacy remain challenges, clinical case reports demonstrate the potential of phage therapy to be lifesaving when conventional treatment fails.

### Microbiome-based and adjunctive strategies

**Figure 3.**
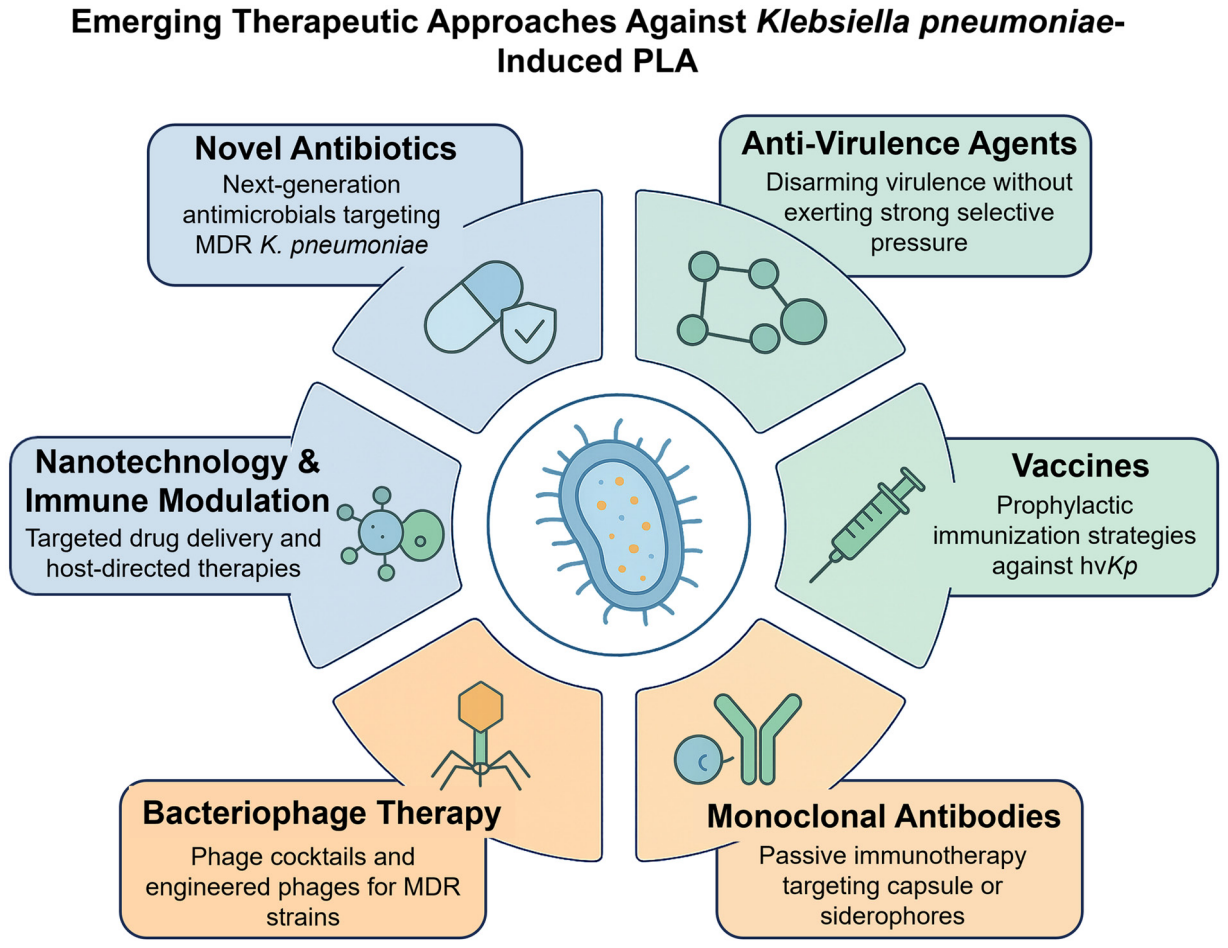
Emerging therapeutic approaches against *Klebsiella pneumoniae*-induced PLA. The central illustration shows hypervirulent, drug-resistant *K. pneumoniae* with capsule, pili and siderophores. Surrounding panels highlight six strategies: Novel antibiotics, anti-virulence agents, vaccines, monoclonal antibodies, bacteriophage therapy and nanotechnology/immune modulation. Colour codes indicate antibiotic-based (blue), immune/vaccine-based (green) and innovative approaches (orange). PLA, pyogenic liver abscess; MDR, multidrug resistance; hvKp, hypervirulent *Klebsiella pneumoniae*.

*K. pneumoniae* is a part of the gut microbiota and its changes may initiate systemic invasion (22). This has sparked interest in microbiome-targeted therapies, including probiotics and faecal microbiota transplantation, to restore microbial balance and reduce hvKp colonization. CRISPR, for example, is being explored not just as a genomic editing tool that works at the gene-targeting level, such as virulence determinants or resistance genes, but also as a programmable antimicrobial system capable of selectively eliminating resistant or hypervirulent bacteria while sparing commensal flora (111). While they are still experimental, these strategies represent new approaches to reduce pathogen load and prevent reinfection. CRISPR-based antimicrobials are also being investigated as precision tools to target resistance or virulence genes in hvKp selectively (103). The management of *K. pneumoniae* PLA involves a combination of antibiotics and drainage; however, new strategies are currently under active investigation, including β-lactam/β-lactamase inhibitors, cefiderocol, vaccines and phage therapy (Table V) (103). Fig. 3 highlights therapeutic strategies against *K. pneumoniae-*induced PLA.

## 8. Future perspectives and clinical challenges

*K. pneumoniae* PLA remains a major clinical challenge despite recent developments in diagnostic modalities and treatment options (14). The emerging epidemiology of this infection is characterised by the convergence of hypervirulence and antimicrobial resistance, which complicates both treatment and prevention measures. In the future, breakthroughs in therapeutics, combined with enhanced clinical and public health approaches, will be key to alleviating the disease burden and improving outcomes (103).

### Convergence of hypervirulence and resistance

During recent years, some of the most concerning findings have involved the emergence of hypervirulent phenotypes that are also multidrug resistant (112). These strains challenge the classic divide between more resistant but less virulent ‘classic’ isolates and more overtly virulent, although antibiotic-susceptible, ‘hypervirulent’ strains. These strains can be considered ‘superbug’ strains as they combine the invasiveness of hvKp with the drug resistance of cKp, eliminating the therapeutic advantage previously afforded by antibiotic susceptibility. Future genomic surveillance and rapid diagnostics will be necessary to ensure these strains are rapidly identified and contained. The clinical manifestation of this convergence is dire; infections due to these pathogens are associated with high morbidity and mortality and are not amenable to treatment with available antibiotics. Future research will focus on understanding the genetic basis of this convergence and the ecological pressures that drive it, as this may inform surveillance, containment and drug development efforts.

### Diagnostic and predictive limitations

Diagnosis is one of the major limiting factors for clinical management, which is crucial for timely and accurate treatment (98). Culture-based methods, which are standard for testing for antimicrobial resistance, are slow, ultimately creating a vicious circle that delays the start of targeted therapy (113). Molecular diagnostics and rapid resistance detection platforms are not yet readily available in numerous clinical settings. In addition, the severity of disease and the risk of metastatic complications cannot be accurately predicted since clinical features and routine laboratory parameters lack specificity. Newer discoveries of biomarkers, genomic profiling and machine learning-based prediction approaches should enable the early identification of high-risk patients and facilitate personalized therapeutic solutions. Recent advances in metagenomic sequencing and rapid MALDI-TOF-based assays have the potential to improve early pathogen identification; however, their widespread implementation remains limited (114).

Recent diagnostic innovations hold considerable promise for improving the management of hvKp-PLA (114). Metagenomic next-generation sequencing of blood or pus can identify pathogens and resistance determinants directly from clinical specimens without the need for culture (115). However, its routine use remains constrained by high cost, turnaround time and bioinformatic complexity. MALDI-TOF mass spectrometry enables species-level identification within hours from cultured isolates and, in some centres, can be adapted for direct specimen testing through rapid extraction workflows (114). In parallel, rapid PCR-based and isothermal point-of-care assays targeting species markers, virulence loci such as iuc and rmpA as well as common carbapenemase genes, including *bla*NDM and *bla*KPC, provide actionable results within a few hours (116). Although each of these modalities has inherent trade-offs in sensitivity, cost and accessibility, an integrated diagnostic approach, combining rapid molecular detection for early guidance with culture and susceptibility testing for definitive management, offers the greatest potential to reduce time to effective therapy in real-world clinical settings. Successful implementation will ultimately depend on local laboratory infrastructure, cost-benefit considerations and integration with antimicrobial stewardship programs.

### Gaps in therapeutic options

The management options for *K. pneumoniae* PLA are limited by poor penetration of drugs into abscess cavities, the rapid emergence of resistance and a lack of targeted regimens for hvKp (33). Antibiotics may not reach adequate levels in the centre of abscesses as they are poorly vascularized (69). Novel delivery methods, such as drug-eluting beads or local catheter infusions, may enhance intra-abscess drug concentrations and warrant further investigation (117). Additionally, there have been no clinical trials specifically for hvKp-PLA, leaving no consensus on optimal empirical therapy. More extensive registries and prospective studies are necessary to determine the optimal antibiotic regimens and durations for different resistance phenotypes.

### Global and regional disparities

The regional distribution of *K. pneumoniae* PLA is not homogenous (118). These hypervirulent strains are most prevalent in East and Southeast Asia, where the majority of the literature and expertise reside (98). By contrast, MDR strains have a longer epidemiological history in North America and Europe, and hvKp-PLA clusters have only recently been described on these continents. This has implications for both research priorities and clinical approaches. For example, in Asia, priorities may centre on vaccines and anti-virulence strategies to curb hvKp and CRKP, whereas in Western countries, emphasis may lie on antibiotic stewardship and infection control. This step requires sharing surveillance data and best practices between countries.

### Toward integrated and preventive approaches

Integrated methods that apply existing and potential novel strategies may represent a promising future for PLA management (14). This encompasses high-risk population prophylaxis (such as diabetes control and potential vaccine usage), rapid diagnostic testing to target therapy, judicious antimicrobial stewardship to limit resistance selection and the deployment of novel therapeutic modalities. If implemented, preventive measures, especially vaccines for the most common capsule types or siderophores, could substantially reduce incidence. Improved global surveillance systems and stronger health infrastructure are crucial for detecting outbreaks and implementing infection control measures promptly (50). A successful response to *K. pneumoniae* PLA will eventually be coordinated across all sectors of clinical medicine, microbiology and public health.

## 9. Conclusions

*K. pneumoniae* PLA has emerged as a significant clinical and public health concern, driven by the dual threats of hypervirulence and escalating antimicrobial resistance. While advances in imaging, interventional drainage and antibiotic therapy have improved outcomes, the increasing prevalence of strains combining MDR with invasive virulence presents an urgent global challenge. Therapeutic options are limited by poor pharmacokinetics, inconsistent efficacy and the absence of standardized regimens for resistant or hypervirulent infections. The future of management will depend on a multipronged strategy. Continued surveillance is necessary to track the evolving epidemiology and the genetic determinants of virulence and resistance. Innovative therapeutics, including novel antibiotics, anti-virulence agents, immunotherapies and bacteriophage-based approaches, offer promise for more effective, targeted interventions. At the same time, advances in diagnostics and predictive modelling may enable earlier recognition of high-risk patients and more personalized care. Preventive strategies, especially vaccines, together with global antimicrobial stewardship and strengthened health systems, will be central to reducing disease burden. Ultimately, addressing *K. pneumoniae* liver abscess requires an integrated vision that bridges clinical medicine, microbiology and public health. Only coordinated global action and sustained translational research can counter this formidable pathogen and its increasingly complex clinical manifestations.

## Figures and Tables

**Table I.** Host risk factors and the mechanistic basis of susceptibility to *K. pneumoniae*-associated PLA.

First author, year	Risk factor	Mechanistic basis	Clinical impact/prevalence	Remarks	(Refs.)
Zhang *et al*, 2025	Diabetes mellitus	Hyperglycemia impairs neutrophil chemotaxis and phagocytosis	Strongest and most consistent risk factor (50–70% of hvKp PLA cases in Asian cohorts)	Independent risk for metastatic complications	(119)
Li *et al*, 2021	Chronic liver disease/cirrhosis	Impaired hepatic innate immunity (Kupffer cell dysfunction)	Increased incidence and poorer prognosis	Often coexists with alcohol use or hepatitis	(70)
Kaur *et al*, 2018	Malignancy	Disease and chemotherapy cause immunosuppression	Higher susceptibility and poorer outcomes	Common in elderly patients	(62)
Pope *et al*, 2019	Immunosuppressive therapy	Reduced innate/adaptive immunity	Risk of severe and relapsing disease	Steroids, biologics and post-transplant	(120)
Thirugnanasambantham *et al*, 2025	No comorbidity	hvKp can overcome intact defences	Up to 30–40% of hvKp PLA cases occur in otherwise healthy individuals	Key feature distinguishing hvKp from cKp	(11)
Al Ismail *et al*, 2025	Microbiome imbalance/colonization	hvKp intestinal carriage increases the risk of translocation	5–10% carriage rates in Asia; lower but rising in Europe/N. America	Emerging risk factor	(4)

hvKp, hypervirulent *Klebsiella pneumoniae*; PLA, pyogenic liver abscess; cKp classical *Klebsiella pneumoniae*.

**Table II.** Geographical distribution, predominant strains and clinical features of *K. pneumoniae*-associated PLA.

Region/country	Predominant strains/serotypes	Major risk factors	Distinctive clinical features	Remarks/trends	(Refs.)
Taiwan, China, South Korea and Singapore	K1 (ST23) and K2 (ST65 and ST86)	Diabetes, no hepatobiliary disease	Monomicrobial PLA and high metastatic rates (endophthalmitis and meningitis)	Endemic focus; earliest recognition	(3,121)
North America and Europe	K1, K2 and emerging ST375	Often in healthy individuals and still common in diabetes	Increasing autochthonous cases	Expanding incidence; community-onset clusters	(118)
South Asia (India and Pakistan)	Mixed strains; including hvKp ST23 and ST11 (CR-hvKp)	Diabetes, cirrhosis and malignancy	Resistant hvKp reported	Rising ESBL/NDM-positive hvKp	(36)
Middle East	Mixed strains (K1, K2 and ST11)	Diabetes and chronic liver disease	Increasing carbapenemase producers (NDM and OXA-48)	Likely underreported	(122)
Africa	Sparse data and sporadic case reports	Unknown	Sporadic reports only	Likely underrecognized; limited data due to lack of diagnostic capacity and surveillance infrastructure	(123)

hvKp, hypervirulent *Klebsiella pneumoniae*; PLA, pyogenic liver abscess; CR-hvKp, carbapenem-resistant hypervirulent *K. pneumoniae*; NDM, New Delhi metallo-β-lactamase; OXA-48, oxacillinase-48.

**Table III.** Major virulence determinants of *K. pneumoniae* in PLA.

First author, year	Virulence factor	Genetic basis	Role in pathogenesis	Clinical relevance	(Refs.)
Zhu *et al*, 2021	Capsule (K1 and K2;) hypermucoviscosity	cps locus; rmpA/rmpA2	Resistance to phagocytosis, inhibition of complement activation leading to serum resistance and enhanced bloodstream survival	Strongly associated with invasive PLA and metastatic spread; vaccine/antibody target	(59)
Monteiro *et al*, 2024	Siderophores (aerobactin, salmochelin, yersiniabactin and enterobactin)	iuc, iro, ybt and ent loci	Iron acquisition; immune modulation; oxidative stress induction	Aerobactin nearly universal in hvKp PLA strains; candidate for anti-virulence therapy	(124)
Clegg and Murphy, 2017	Fimbrial adhesins (Type 1 and 3 fimbriae)	fim and mrk operons	Colonization of gut; adherence in liver; biofilm formation	Facilitate gut-to-liver translocation	(20)
Miller *et al*, 2024	Lipopolysaccharide	waa gene cluster	Resistance to serum killing; immune modulation	Contributes to serum survival and sepsis severity	(105)
Liao *et al*, 2024	Virulence plasmids	pLVPK-like plasmids (~200 kb)	Encode capsule regulators (rmpA/rmpA2) and siderophores	Horizontal transfer spreads hypervirulence traits	(125)
Lam *et al*, 2018	Pathogenicity islands	ICEKp elements (ybt and clb)	Iron scavenging (ybt) and genotoxicity (colibactin)	Colibactin linked to DNA damage and possible cancer risk	(126)
Bossuet-Greif *et al*, 2018	Colibactin	clb genes on ICEKp	Genotoxic effects and DNA damage in host cells	Recently linked to colorectal cancer; under study	(127)

hvKp, hypervirulent *Klebsiella pneumoniae*; PLA, pyogenic liver abscess; pLVPK, large virulence plasmid of *Klebsiella pneumoniae*; ICEKp, integrative and conjugative element of *Klebsiella pneumoniae*.

**Table IV.** Major antimicrobial resistance mechanisms in *K. pneumoniae* and their clinical implications.

Resistance mechanism	Genetic basis	Antibiotic classes affected	Clinical relevance
Extended-spectrum β-lactamases	*bla*CTX-M, *bla*SHV and *bla*TEM	Third generation cephalosporins	Undermines cephalosporin therapy; common in Asia (30–50%)
Carbapenemases	KPC, NDM, OXA-48 and VIM	Carbapenems	Critical driver of CR-hvKp; global spread
Porin loss (OmpK35/36)	Chromosomal mutations	β-lactams and carbapenems	Enhances β-lactamase resistance
Efflux pumps (AcrAB-TolC)	Regulatory mutations	Fluoroquinolones and tigecycline	Contributes to MDR profiles
Aminoglycoside resistance	AMEs and armA methyltransferase	Aminoglycosides	Limits the use of amikacin/gentamicin
Colistin resistance	mcr plasmid genes and pmrA/B mutations	Colistin (last line)	Increasingly reported, especially in Asia and the Middle East
Fosfomycin resistance	*fosA* gene (plasmid-borne)	Fosfomycin	Emerging, limits use in urinary/liver infections

blaCTX-M, β-lactamase gene encoding cefotaximase-Munich type extended-spectrum β-lactamase; blaSHV, β-lactamase gene encoding sulfhydryl variable type ESBL; blaTEM, β-lactamase gene encoding Temoneira-type ESBL; KPC, *Klebsiella pneumoniae* carbapenemase; NDM, New Delhi metallo-β-lactamase; OXA-48, oxacillinase-48 type carbapenem-hydrolyzing class D β-lactamase; VIM, Verona integron-encoded metallo-β-lactamase; CR-hvKp, carbapenem-resistant hypervirulent *Klebsiella pneumoniae*; MDR, multidrug resistance; AMEs, aminoglycoside-modifying enzymes; OmpK35/36, outer membrane porin proteins K35 and K36.

**Table V.** Current and emerging therapeutic strategies for *K. pneumoniae* PLA.

Strategy	Mechanism of action	Advantages	Limitations	Clinical status
Cephalosporins (Third generation)	Inhibit cell wall synthesis	Effective if susceptible	ESBL undermines efficacy	Routine use; declining efficacy in ESBL-prevalent regions
Carbapenems	Broad-spectrum β-lactams	Active vs. ESBL strains	Resistance spreading	Standard therapy; limited
Tigecycline and Colistin	Ribosomal inhibition/membrane disruption	Salvage for MDR	Poor penetration, toxicity	Last-line therapy
Fosfomycin	Cell wall inhibition (MurA)	Active vs. some MDR	fosA resistance is common	Off-label use
Novel β-lactam/β-lactamase inhibitors (such as ceftazidime-avibactam, meropenem-vaborbactam and imipenem-relebactam)	Inhibit β-lactamases	Effective vs. KPC/OXA-48	Limited vs. NDM	Approved for systemic infections; limited clinical data in PLA
Cefiderocol (siderophore cephalosporin)	Exploits iron uptake pathways	Potent vs. MDR/CRKP	Resistance may develop; limited liver data	Approved; clinical use expanding
Eravacycline and Plazomicin	Ribosomal inhibition (tet)/next-generation aminoglycoside	Activity vs. MDR strains	Limited PLA-specific data	Approved, limited use
Anti-virulence agents	Block capsule or siderophores	Low resistance pressure	Preclinical stage	Experimental
Vaccines (K1/K2 and siderophore-based)	Prevent capsule/siderophore infection	Prophylaxis potential	Serotype diversity	Preclinical/early clinical
Monoclonal antibodies	Neutralize capsule, LPS and siderophores	Adjunctive therapy	Expensive; narrow targets	Preclinical/early
Bacteriophage/phage enzymes	Lytic activity vs. MDR hvKp	Active vs. CR-hvKp	Specificity, regulatory hurdles	Case reports, trials emerging
Microbiome interventions (probiotics and FMT)	Restore gut balance, reduce hvKp colonization	Prevent recurrence	Experimental; early preclinical data only	Preclinical

PLA, pyogenic liver abscess; MDR, multidrug resistance; ESBL, extended-spectrum β-lactamase; KPC, *Klebsiella pneumoniae* carbapenemase; hvKp, hypervirulent *Klebsiella pneumoniae*; CRKP, carbapenem-resistant *Klebsiella pneumoniae;* NDM, New Delhi metallo-β-lactamase.

## Data Availability

Not applicable.
